# Enhancement of Adhesive Bonding Properties of Polyetheretherketone-based Materials using Plasma Surface Modifications

**DOI:** 10.3290/j.jad.b2838149

**Published:** 2022-03-24

**Authors:** Canan Akay, Natiga İsrafil, Suat Pat

**Affiliations:** a Associate Professor, Department of Prosthodontics, Faculty of Dentistry, Osmangazi University, Eskişehir, Turkey. Idea, hypothesis, experimental design, performed the experiments, wrote the manuscript.; b Research Assistant, Department of Prosthodontics, Faculty of Dentistry, Osmangazi University, Eskişehir, Turkey. Experimental design, performed the experiments.; c Professor, Department of Physics, Eskişehir Osmangazi University, Eskisehir, Turkey. Experimental design, performed the experiments.

**Keywords:** polyetheretherketone, surface treatment, shear bond strength, surface roughness, veneering procedure, adhesion in dentistry

## Abstract

**Purpose:**

To investigate the effects of plasma surface treatments and methyl methacrylate-based adhesives on polyetheretherketone.

**Materials and Methods:**

One hundred ten polyetheretherketone specimens were fabricated and divided into five pretreatment groups: group ArP, 100% argon plasma; group ArOP, 50% argon + 50% oxygen plasma; group ArNP, 50% argon + 50% nitrogen plasma; group ArONP, 75% argon + 12.5% oxygen + 12.5% nitrogen plasma; group C, control. Atomic force microscopy and scanning electron microscopy were performed after surface treatments. After topographical surface examinations, Visio.link primer (Bredent) (n = 10) was applied to the surface of half of the samples in each group (n = 20) and the veneering resin was polymerized onto the polyetheretherketone. The shear bond strengths were measured using a universal test machine.

**Results:**

The mean bond strengths of the Visio.link primer applied to group ArP and group ArONP (13.9 and 13.6 MPa, respectively) were statistically significantly higher than that of group C (9.0 MPa). The average shear bond strength of the Visio.link subgroups was higher than that of the Visio.link subgroups (p > 0.05).

**Conclusions:**

The use of a methyl methacrylate-based adhesive (Visiolink) provides bonding between polyetheretherketone-veneering composites. Different plasma treatments without primer application had no significant effect on bonding.

Polyetheretherketone (PEEK) is a polymer from the polyaryletherketone (PAEK) group, a new high-temperature thermoplastic polymer family consisting of an aromatic basic structure and molecular chain that links ketone and ether functional groups.^4,6,18,21,22,23^ PEEK is one of the few polymers used instead of metals in many fields. In the medical field, it is used in spine surgery, intervertebral discs, finger prostheses, fixation of fractures, maxillofacial surgery, and joint prostheses.^14^ In dentistry, PEEK can be used in removable and fixed partial denture framework designs, implants, temporary abutments for implant-supported prostheses, healing abutments, implant-supported bars, orthodontic wires, and space maintainers in pedodontics.^2,22,25,27^

PEEK exhibits excellent mechanical properties at high temperatures. It is a hard, opaque material with unique properties such as resistance to abrasion, electrical and temperature resistance, good dimensional stability at high temperatures, and machinability. This material can be easily used in preparing partial denture frameworks with a digital design suited to the patient’s anatomy. Since prosthetic frameworks do not contain metals, they do not create a metallic taste in the mouth. It has no thermal or electrical conductivity, and has a radiolucent appearance. It is nonallergenic, durable, and light. PEEK frameworks absorb stress during chewing. It shows a high resistance to abrasion. In addition, it has a high melting point (approximately 343°C) and its chemical stability when in contact with almost all organic and inorganic chemicals make it an interesting material for metal-free prosthodontics, as well as being biocompatible.^6,22,25^

Thus, PEEK is a useful material for dental applications. However, the grayish-white color and low translucency of PEEK limits its use as a monolithic tooth restoration material.^23^ Therefore, additional veneering is required to achieve satisfactory esthetics. However, to provide sufficient functional results and long-term stability, durable bonding between PEEK and veneers should be provided.^22^ Bonding can be created through chemical or micromechanical retention, or a combination thereof. It depends on the composition and interaction of the materials used.^20^ In scientific studies, PEEK materials with ceramic fillers of different concentrations are most commonly used, PEEK materials with glass, silicone, and carbon fillers have not been studied as extensively.^27^ In one study, argon-oxygen (Ar:O_2_) plasma-treated filler-free PEEK showed higher shear bond strength than PEEK materials with 20% titanium dioxide (TiO_2_), 30% carbon, and other contents and proportions of fillers.^20^ When no surface treatment is performed on the PEEK surface, it creates insufficient bonding with resin materials owing to its chemical composition and low surface energy.^22,23^ Many material properties such as adhesive bonding properties, wettability, friction coefficient, and surface roughness can be significantly affected by surface treatment.^12^

In addition, the positive contribution of the primers to a stronger link between PEEK and composite veneers has been reported.^23^ Among these agents, Visio.link primer (Bredent), which contains methyl methacrylate (MMA), was found to be more effective.^2,12,24^ Plasma treatment is used as an alternative to overcome the disadvantages of conventional sandblasting or chemical wear. The potential effect of sandblasting, which is a less expensive method, depends on the distance and angle of application.^15^ The pressure is variable and depends on the operator, and it is sensitive to particle size and treatment time.^27^ Application of high-strength concentrated acids, such as sulfuric acid or piranha solution, in PEEK surface treatments can cause chemical damage. Piranha solution (peroxymonosulfuric acid, H_2_SO_5_) consists of a 10:3 mixture of 98% H_2_SO_4_ and 30% hydrogen peroxide (H_2_O_2_). Therefore, they are considered unsafe in clinical and laboratory settings.^12,19^

Plasma application increases the surface energy of different materials and increases the bond strength between polymer materials and other materials. As a result of the low penetration level, plasma modification maintains the physical and chemical properties of the material mass, thereby improving the surface quality.^5,21^

This study aimed to investigate the effects of plasma surface treatments with argon, argon-oxygen, argon-nitrogen and argon-oxygen-nitrogen gases and adhesive material application on the shear bond strength between PEEK and veneering composites.

## Materials and Methods

### Specimen Preparation

The manufacturers and compositions of the materials used in this study are shown in Table 1.

**Table 1:** Composition and manufacturers of the materials used in the study

Material	Manufacturer	Chemical composition	Lot No.
PEEK blank (CopraPeek light) 98 mm x 10 mm	Whitepeaks; Essen, Germany		E 10002
Primer material (Visio.link)	Bredent; Senden, Germany	Methyl methacrylate (MMA), penta-erythriol-triacrylate (PETIA), light activator	171936
Autopolymerizing acrylic resin (Integra)	United Dental Group (IMG); Ankara, Turkey	95% methylmethacrylate (MMA), 5% ethylene glycol dimethylacrylate (EGDMA)	170517
Composite veneer material (G-ænial Posterior A3)	GC; Tokyo, Japan	Methacrylate monomer, silica, strontium and lantanoid fluoride prepolymerized filler	180904A

One hundred ten rectangular (10 x 5 x 2 mm^3^) prism-shaped samples were milled from the PEEK block. These samples were embedded in an auto-polymerizing acrylic resin, and the bonding surfaces of each sample were polished with 800-grit, 1000-grit, 1200-grit and 1500-grit silicon carbide papers in a polishing machine under running water for 30 s (Meta Serv 250 Buehler; Lake Bluff, IL, USA) to obtain a standardized surface. Then, polished samples were ultrasonically cleaned in a distilled water bath for 15 min and air dried before surface modifications.

### Surface Modifications

Samples were randomly divided into five groups according to surface modification procedures. PEEK surfaces were modified with one of these five surface modification methods.

- Group ArP: 100% argon plasma (Dressler Cesar 136 RF Generator, Advanced Energy Industries; Ft. Collins, CO, USA) was applied on PEEK surfaces for 35 min at vacuum chamber pressure of 789 Torr (10^-6^ atm), with 13.56 MHz radiofrequency (RF) power supply.
- Group ArOP: mixture of 50% argon and 50% oxygen plasma (Dressler, Cesar 136) was applied on PEEK surfaces for 35 min at vacuum chamber pressure of 789.10^-3^ atm with 13.56 MHz RF power supply.
- Group ArNP: mixture of 50% argon and 50% nitrogen plasma (Dressler, Cesar 136) was applied on PEEK surfaces for 35 min at vacuum- chamber pressure of 789 Torr (10^-6^ atm) with 13.56 MHz RF power supply.
- Group ArONP: mixture of 75% argon, 12.5% oxygen and 12.5% nitrogen plasma (Dressler, Cesar 136) was applied on PEEK surfaces for 35 min at vacuum chamber pressure of 789 Torr (10^-6^ atm), with 13.56 MHz RF power supply.
- Group C: nontreated PEEK surface group served as control.

### Topographical Analysis

To analyze their morphological features, the surface-modified PEEK samples from each group were cleaned with 96% ethanol and air dried. A sample from each surface-modification group (n = 5) was selected for SEM (Hitachi Regulus 8230 FE-SEM; Tokyo, Japan) with 20 kV and 11.1 mm. Before analysis, PEEK surfaces were sputter-coated with gold for 2 min using a gold-plating device (Leica EM ACE600; Wetzlar, Germany), then attached to metal stubs using carbon tape. The specimens were positioned inside a high-resolution field-emission scanning electron microscope (FE-SEM), and images of the samples at 10,000X 20,000X original magnification were recorded on the FE-SEM.

### Surface Roughness Measurement

Atomic force microscopy (AFM-Veeco, Multimode V; Plainview, NY, USA) was also used to analyze the morphology of the surface-modified PEEK samples. The images were captured under normal ambient conditions. The observations were carried out in tapping mode using a 1 to 10 Ω-cm phosphorus-(n) doped Si tip. Depending on the vertical position of the probe tip, the heights of the surfaces were recorded as either bright or dark in the generated images.

### Bonding Procedures

Twenty samples in total were obtained for each surface modification method (n = 20), and the samples were randomly divided into two subgroups (n = 10) to determine shear bond strengths for the veneering resin. In each group, half of the PEEK surfaces were coated with an adhesive, which was polymerized using a polymerization unit (Planmeca Unit; Helsinki, Finland). The adhesive was polymerized at 220 mw/cm^2^ and a wavelength range of 370–400 nm for 30 s with the device set for 30 s in linear curing mode. Then, all specimens (n = 10) were veneered with a composite resin (2 mm diameter, 2 mm thick) using a teflon mold (Ultradent; South Jordan, UT, USA) for standardization; the veneering resins were polymerized using the same polymerization unit for 20 s.

### Shear Bond Strength Measurements

The shear bond strength of the PEEK core/veneering resin interface was measured using a universal testing machine (Mod Dental; Ankara, Turkey). The specimens were fixed in a special jig, with the loading tip close to the bonding surface. The load was applied with a flat bladed blade at a crosshead speed of 0.5 mm/min, with the bonding surface parallel to the loading tip. The maximum load at debonding was measured. The shear bond strengths were calculated using the formula σ =F/S (σ = shear bond strength, F = the load [N] at failure, S = surface area of the PEEK core/veneering resin interface [mm^2^]).

### Failure Mode Analysis

The fractured surface of the specimens was observed and the failure types described as: type 1: adhesive failure (< 20% composite resin observed at the PEEK surface); type 2: cohesive failure (more than 80% composite resin observed at the PEEK surface); type 3: mixed failure (20%–80% composite resin observed at the PEEK surface).

### Statistical Analysis

All statistical tests were conducted using SPSS Statistics 21.0 (IBM SPSS; Armonk, NY, USA). The Shapiro-Wilk test was used to test the data for normal distribution. Bilateral variance analysis was used to compare the bond strength averages of subgroups with and without Visio.link in each group, and with subgroups without Visio.link between groups. With the Bonferroni multiple comparisons test, a statistical comparison of the subgroups in which Visio.link was applied was performed. Statistical significance was set at p < 0.05.

## Results

In ArP, ArNP and ArONP groups, the shear bond strength between PEEK and the veneering composite was relatively high. The shear bond strengths obtained for PEEK samples are shown in Table 2.

**Table 2:** Bond strengths (MPa) and failure mode distributions

Groups	Bond strength by surface treatment (MPa), mean ± SD	Failure mode		
		Adhesive	Cohesive	Mixed
Control subgroups ArP	2.6 ± 0.3^A^	10	–	–
Control subgroups ArOP	3.0 ± 0.4^A^	10	–	–
Control subgroups ArNP	2.8 ± 0.3^A^	10	–	–
Control subgroups ArONP	2.8 ± 0.3^A^	9	–	1
Control subgroups C-Control	1.1 ± 0.3^A^	10	–	–
Visio.link subgroups ArP	13.9 ± 4.0^C^	9	–	1
Visio.link subgroups ArOP	7.0 ± 1.6^B^	9	–	–
Visio.link subgroups ArNP	11.6 ± 3.7^C^	10	–	–
Visio.link subgroups ArONP	13.6 ± .0^C^	9	–	1
Visio.link subgroups C-Control	9.0 ± 1.6^B^	10	–	–

Different superscript letters indicated significant differences between groups (p < 0.05).

Two-way ANOVA, which compared all groups within themselves according to their bond strength means, showed a statistically significant difference between subgroups in which Visio.link was applied or not applied (control) (Table 2).

A statistically significant difference was observed between the control and Visio.link subgroups of ArP, ArOP, ArNP, ArONP. A statistically significant difference was observed between the control and Visio.link subgroups (p = 4.88 x e-10) (p < 0.001).

The comparisons between groups showed no statistically significant difference between the subgroups without Visio.link (control) in terms of shear bond strengths (Table 2) (p > 0.05).

### Failure Analysis

The failure analysis of each group is given in Table 2. Almost all samples exhibited adhesive failure.

### SEM Evaluation

**Fig 1:**
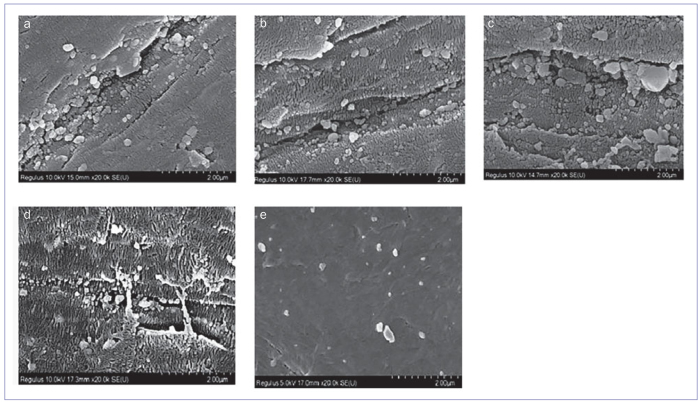
FE-SEM images 20,000X original magnification; a: group ArP; b: group ArOP; c: group ArNP; d: group ArONP; e: group C.

SEM images of the PEEK surface samples are shown in Fig 1. Fine scratches and relatively few microtubules were found in the ArP group at 20,000X original magnification (Fig 1a). The SEM images of the ArOP group presented a significantly greater number of small scratches and knots compared to the ArP group (Fig 1b). In the ArNP group, a large number of microplates were observed around the pits across the entire surface (Fig 1c). In the ArONP group, more regular and irregular scratches, prominent grooves, and cracks were observed on the surface than was the case in the other groups (Fig 1d). Since no surface treatment was performed in the control group, and very few small scratches were seen in the SEM images, a regular surface morphology was observed which approached smoothness (Fig 1e). Except for the highest roughness observed in the ArONP group and the least roughness in group C (Fig 1), the SEM images of PEEK samples did not display much difference between the groups.

### AFM Evaluation

**Fig 2:**
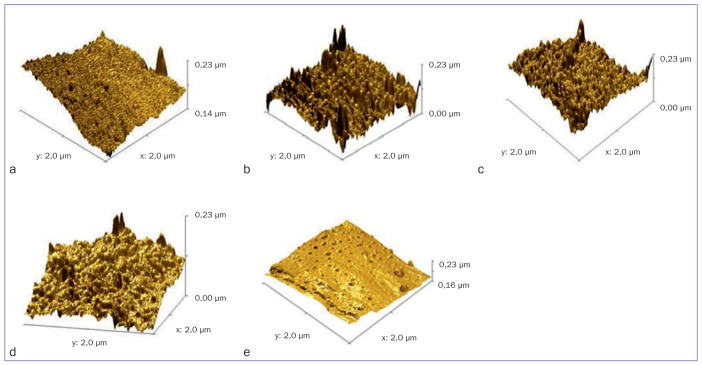
AFM findings. a: group ArP; b: group ArOP; c: group ArNP; d: group ArONP; e: group C.

In addition to the SEM analysis, which visualizes PEEK surface roughness at various magnifications, AFM examinations were also performed to express roughness numerically. The average surface roughness (Ra) obtained varied between the groups (Table 3 and Fig 2). The N content increased the Ra value compared to the control specimens and other specimen groups. Oxygen gas increased the Ra values compared to control specimens, but it changed only slightly according to the other plasma treatment specimens. Ra values for all plasma treatment samples increased compared to the control samples.

**Table 3:** Average roughness (Ra) values in individual groups in a 2-µm and 4-µm image

Average roughness (Ra) values	Groups				
	ArP	ArOP	ArNP	ArONP	C
2-µm image	6.73 nm	8.94 nm	13.1 nm	9.65 nm	5.80 nm
4-µm image	14.6 nm	13.1 nm	29.1 nm	14.9 nm	10.8 nm

## Discussion

This in vitro study evaluated the effect of plasma surface modification and application of MMA-based adhesives on the shear bond strength of veneering composites to PEEK. The mean bond strengths of the Visio.link primer applied in the ArP and ArONP groups (13.9 and 13.6 MPa, respectively) were statistically significantly higher than that of the control group (9.0 MPa). The average shear bond strengths of the Visio.link subgroups were higher than those of the subgroups in which Visio.link was not applied.

By providing the desired chemical bond between PEEK and veneering composites, the difficulty arising from the low surface energy of PEEK is eliminated.^13^ Several different surface-modification methods have been investigated in the past, primarily based on mechanical modification of the surface. Plasma surface modification is applied in many areas of medicine.^7,16^ Plasma is the third physical state of matter, which consists of ionized gas particles. Apart from electrically charged particles, plasma produces ultraviolet and thermal radiation, visible light, and reactive molecules,^7^ whose composition can vary according to the plasma characteristics. The main surface changes caused by plasma treatment are increased surface wettability, altered surface roughness, and generation of radicals thanks to ultraviolet radiation.^1^

Plasma has different effects according to its type, application power, duration, and type of gas used.^8,26^ Plasma can be used at atmospheric or low pressures. Methods based on atmospheric plasma treatments have the advantage of ease of use, as they can be applied without requiring a vacuum. Throughout the process, the material is in contact with the atmosphere. The surface can react with atmospheric and plasma gases. In atmospheric plasma device applications, an increase in the plasma effect is expected.^5^ Various atmospheric pressure plasma applications have been shown to be effective in improving the adhesion quality of the polymer surface.^3,17^

It has been stated that atmospheric pressure plasma is more effective in increasing the surface energy of PEEK than low pressure plasma. Plasma application has a varying effect on increasing adhesion by creating some chemical groups, eg, Ar+O_2_ /Ar+N_2_/Ar+O_2_+N_2_. When polymers are exposed to gas plasma, two important chemical processes are expected to occur on the surface. First, plasma ions bombard polymer surfaces and break polymer chains, and second, they form small degradation products.^9,11^

Zhou et al^29^ evaluated the bond strength between PEEK specimens and the veneering composite material after applying different surface treatments (sandblasting, Ar plasma, femtosecond laser) and aging procedures. The highest bond strength was observed for the Ar plasma group (8.5 MPa). In the present study, the bond strength of the ArP group (13.86 ± 4.04 MPa) was higher than that in a similar study.^29^ Different aging procedures reduce bond strength, but the present study did not subject PEEK samples to aging.

Zhou et al^30^ assessed the effect of different surface treatments (sandblasting, sulfuric acid, hydrofluoric acid, and Ar plasma) on the bond strength between PEEK and the veneering composite. They found that the bond strength was 6.8 MPa in the argon plasma-treated group; the highest bond strength was observed in the sulfuric acid (8.7 MPa) group. The reason for the high bond strength (13.9 ± 4.0 MPa) in the ArP group in the present study was the use of Visio.link primer, which contains MMA.

Schwitalla et al^20^ obtained higher bond strengths, especially in PEEK samples without fillers, as a result of surface treatment with low-pressure Ar:O_2_ plasma. The bond strength was 7.63 MPa in the plasma-treated group and 19.8 MPa in the plasma-treated group with sandblasting. Although the bond strength (7.0 MPa) of the ArOP group in our study was close to that of the plasma-treated group in the previous study,^20^ it was lower than that of the plasma-treated group with sandblasting. This can be explained by the positive effect of sandblasting on bond strength and surface roughness.^20^

Failure mode analysis can help explain the bond strength results. All subgroup specimens revealed debonding at the PEEK interface. Moreover, there were almost no remnants of composite resins on the surfaces of PEEK, suggesting a probable lack of adherence to PEEK surfaces, even though they were surface-modified.

The plasma forms active chemical groups that generally contain oxygen on the polymer (PEEK) surface. The 50% Ar + 50% O_2_ (group ArOP) plasma in this study further increased the active chemical groups and the bond strength. In our study, the average bond strength (7.0 MPa) was unexpectedly low in all groups in the Visio.link ArOP group. In a study by Zhang et al,^28^ when the PEEK surface was treated with low-pressure Ar, O_2_, and N_2_ plasmas without applying primer, bond strengths were also low, with Ar plasma yielding 1.3 MPa, O_2_ plasma 1.1 MPa, and N_2_ plasma 1.0 MPa. Zhang et al^28^ also reported that the overall oxygen content, density of the C–O groups, and polarized surface energy positively affected the bond strength between materials, whereas C=O groups negatively affected the bond strength. Considering this, we can explain why the Ar:O_2_ plasma in our study led to a low average bond strength. Since different oxygen groups may occur that affect the surface bond strength positively or negatively, O_2_ plasma may not increase bond strength sufficiently or at all. In our study, the mean bond strength (13.60 ± 3.0 MPa) in the ArONP group, which also includes O_2_ gas, was the second-highest among the groups after Ar plasma treatment. This is thought to be related to the high efficacy of Ar gas in the plasma mixture. In our study, unlike the study by Zhang et al,^28^ the ArNP group (11.6 ± 3.7 MPa) with Visio.link and N_2_ plasma had a higher bond strength than did the ArOP group (7.0 ± 1.6 MPa), which included oxygen. This may be due to the use of 100% O_2_ and 100% N_2_ in the plasma groups in the previous study.

In this study, the mean shear bond strength ranged from 7.0 to 13.9 MPa in all subgroups in which Visiolink was applied. Mean shear bond strengths between PEEK and veneering composite were found to be 1.1–3.0 MPa in subgroups in which Visiolink was applied. This does not meet ISO standards, because the mean bond strength was less than 5 MPa.^10^

## Conclusion

Veneering composites should not be applied to PEEK substructures without primers, since bond strengths were below ISO standards in all subgroups without primer (Visio.link). The ArP and ArONP groups had higher PEEK-veneering composite bonding values.

Surface topography is an important factor affecting the micromechanical bonding between PEEK and composite materials. According to the results of the AFM and SEM analyses, Ar, N_2_, and O_2_ plasma applications created a rougher surface on the PEEK surface compared to that in the control group. AFM showed that the ArNP group had the highest surface roughness value (29.1 nm).

## Clinical relevance:

Primer and plasma applications increase the bond strength of the coating composite to be applied to the PEEK surface. In this way, it can be safely used as a substructure material in fixed prostheses in the clinic.
